# The pattern of change in opioid and adjuvant prescriptions for cancer pain before and after referral to a comprehensive program in the Palliative Care Center in Kuwait

**DOI:** 10.1186/s12904-021-00717-2

**Published:** 2021-02-03

**Authors:** Ameena Mohammed Al-Ansari, Wafaa Mostafa Abd-El-Gawad, Sobhi Mostafa AboSerea, Eman El Sayed ElShereafy, Fatma Abdel Shakor Ali, Mohammed Abd Elaziz ElSayed

**Affiliations:** 1 Palliative Care Center, Al-Sabah Medical Area, Al-Shuwaikh, Kuwait; 2 Geriatrics and Gerontology Department, Faculty of Medicine, Ain Shams University, Al- Abbaseya, Cairo, Egypt

**Keywords:** Palliative care center, Kuwait, Cancer-related pain, Morphine equivalent daily dose, Opioids, Adjuvant medications, Anxiety, Depression

## Abstract

**Background:**

Cancer-related pain is a complicated symptom that often coincides with fatigue, depression, and anxiety. Although many safe treatments are available, inadequate control of Cancer-related pain continues to lead to suffering in cancer patients. This study’s aim is to describe pain control, and the pattern of change in opioid and adjuvant medication prescriptions, before and after referral to the Palliative Care Center.

**Methods:**

We conducted a prospective cohort study in adult cancer patients the Palliative Care Center between January 1, 2016 and December 30, 2017. We measured pain intensity and other associated symptoms via the Revised Edmonton Symptom Assessment System (ESAS-r) and documented detailed analgesics and adjuvant medication history before starting any palliative care and on days 0, 3, 6, and 14.

**Results:**

The analysis included 240 patients whose cancer-related pain, anxiety, and depression scores meaningfully improved by day 6. The changes in the median (interquartile ranges) of Cancer-related pain, anxiety, and depression scores from day 0 to day 6 were: 6 (4–8) to 3 (1–4); 6 (4–9) to 2 (1–4); and 3 (2–6) to 2 (1–4), respectively, with *p* < 0.001 for all. Morphine was the most common opioid administered; the percentage using it increased from 20.4% (*n* = 49) before referral to 49.6% (*n* = 119) on day 6 (*p* < 0.001). The median morphine equivalent daily dose decreased from a median (interquartile ranges) of 60(31–93) mg/day before referral to 34(22–66) mg/day on day 6 (*p* < 0.001). There was also a statistically significant increase in the percentage of patients taking adjuvant medications, from 38.8% before referral to 84.2% on day 6 (*p* < 0.001). Comparing D0 to D6, the number of patients using Gabapentinoids significantly increased from 57(23.75%) to 79(32.9%) (p < 0.001), amitriptyline dramatically increased from 14 (5.8%) to 44 (18.3%) (p < 0.001), and other antidepressant drugs increased from 15 (6.2%) to 34 (14.1%) (p < 0.001).

**Conclusion:**

After referral to the Palliative Care Center, patients’ pain and other symptoms scores decreased significantly, even with lower median morphine equivalent daily doses, arguably through more appropriately directed opioid use. This is evidence for the effectiveness of the comprehensive program at the Palliative Care Center in Kuwait.

**Supplementary Information:**

The online version contains supplementary material available at 10.1186/s12904-021-00717-2.

## Background

Cancer-related pain (CRP) is a common symptom in cancer patients. It can relate directly to either malignancy or anticancer treatment. In a systematic review of 52 studies, the prevalence of CRP was 59% in patients undergoing anticancer therapy, and 64% in those with advanced or metastatic disease [1, 2]. Undoubtedly, unrelieved pain can interfere with physical functioning and one’s quality of life, which may decrease the patient’s adherence to anticancer therapy or eventually lead to suffering in the terminal phase [3, 4].

Guidelines published by various organizations, such as the World Health Organization [5, 6], the European Association for Palliative Care [7], the European Society of Medical Oncology [8], and, recently, the American Society of Clinical Oncology [9], have emphasized the importance of the appropriate assessment and management of CRP, including through the use of opioids [5–11].

Although guidelines exist, and several relatively safe treatment modalities are available, many cancer patients are still suffering from inadequate CRP control [12]. A systematic review that included 26 studies reported that the prevalence of CRP undertreatment had reached 82% [13]. In a more recent review of 20 studies published in 2014, the prevalence of undertreatment had decreased to 31.8%, but remained relatively high [14].

CRP is a complicated symptom that is frequently reported alongside other symptoms such as fatigue, depression, and anxiety. The relationship between pain, anxiety, and depression is complex and bidirectional [15–18]. Coexisting depression or anxiety may increase the complexity and difficulty of pain management [15, 16]. Similarly, inadequate pain control can lead to an increased prevalence and severity of these other symptoms [17, 18].

The role of adjuvant medications in CRP management is debatable. Many studies have successfully used combinations of adjuvant analgesics, with and without opioids, to manage the majority of cancer pain [19, 20]. The rationale for combining opioids with adjuvant analgesics includes: improved pain control relative to opioids alone, lower opioid doses, and potentially reduced opioid-related side effects [21, 22]. On the other hand, many studies have demonstrated no benefit from adjuvant medications [23, 24].

Addressing patients’ in-depth, physical, emotional, and spiritual suffering is the core of palliative care. Therefore, to improve the quality of life in cancer patients, the early integration of palliative care with oncology is highly recommended [25, 26].

A palliative care team’s interdisciplinary approach focuses on the impeccable assessment of total pain by taking a holistic approach to CRP management [15, 16].

To the best of our knowledge, no one has well documented CRP control before and after referral to the Palliative Care Center (PCC) in Kuwait. Given the limited amount of published data from Kuwait, we consider this study a benchmark for determining the adequacy of CRP management.

The PCC is a specialized tertiary medical center that focuses on treating the complex symptoms and quality of life of cancer patients. A multidisciplinary team cares for the patients. The head of the team is a specialist in palliative medicine, and the other members are physicians with palliative care training, primarily from internal medicine or oncology, nurses, nutritionists, physiotherapists, psychosocial workers, and spiritual advocates. Outpatient clinics, inpatient admissions, and ambulatory consultations of PCC are covering all patients in Kuwait.

The primary aim of this study was to describe patients’ pain control, and the pattern of changes in their opioid and adjuvant medication prescriptions, before and after referral to the PCC.

## Methods

### Setting and design

We conducted a prospective cohort study of all new patients referred to the PCC in Kuwait from January 1, 2016 to December 30, 2017.

### Patient selection

#### Inclusion and exclusion criteria

We enrolled all adult patients (18 or older) with cancer diagnoses and CRP, referred to either the inpatient or the outpatient clinic of the PCC, in the study. Patients were eligible if they were able to rate their pain and other symptoms and provided informed consent to participate in the study. We excluded patients if they were younger than 18 years old, had no evidence of cancer or CRP, could not describe their pain, refused to participate, or who went missing during follow-ups.

### Assessment data and tools

The study collected demographic data regarding patient age, sex, nationality, as well as data on their clinical history, including cancer diagnosis, metastasis, documented active anticancer treatment or palliative status, pain characteristics, and referring health care facility. This study defined CRP as any pain that could be anatomically or physiologically linked to cancer or its treatment. The study included mild, moderate, and severe pain, either intermittent or continuous. In addition to the clinical assessment of pain, we utilized the Leeds Assessment of Neuropathic Symptoms and Signs (LANSS) pain scale. It comprises two parts: the first part is pain history over the prior week, and the second part is a sensory examination. It discriminates neuropathic pain from nociceptive pain [27]. The LANSS scale is a useful, validated tool for classifying pain in cancer patients that has a sensitivity of 86% and specificity of 100% [28].

The study measured pain intensity and other associated symptoms using the Revised Edmonton Symptom Assessment System (ESAS-r) during the first consultation, before starting any palliative care plan (D0), and then again on Day 3 (D3), Day 6 (D6), and Day 14 (D14).

#### Revised Edmonton symptom assessment system (ESAS-r) [29–31]

The ESAS is a validated tool for the assessment of symptoms in a palliative care setting. Patients rate the severity of their symptoms over the previous 24 h from 0 (no symptom) to 10 (worst). The ESAS has a high test-retest reliability of > 0.8 and has been validated in many clinical settings, particularly in cancer patients [29, 32].

The ESAS has consistently been used to assess anxiety and depression. It has a good association with the Hospital Anxiety and Depression Scale [33]. We routinely employed the DSM-V criteria to diagnose anxiety and depression in the patients, often on the first or second visit, and repeated if necessary, during follow-ups [34].

The ESAS has been revised to improve the ease with which patients can understand and complete it. Because of the diversity of Kuwait culture, which includes more than ten nationalities, we used validated English and Arabic versions [31].

#### Edmonton classification system for Cancer Pain [35]

This is the most-validated cancer pain classification system, and it enables standardized reporting based on the ESAS score. It allows both clinicians and researchers to use a common language in or between various different clinical settings [36]. This system classifies pain as either mild (0–3), moderate (4–6), or severe (7–10) [35–37].

#### Detailed history of pain medications

We recorded detailed histories of the patients’ opioid and non-opioid analgesics and adjuvant medications, including their type, dose, route, regularity, frequency, and as-required doses in the 24 h before or after referral on D0, D3, and D6. We calculated the oral morphine equivalent daily doses (MEDDs) according to standard recommendations [38, 39].

#### Pain management Index [40, 41]

The pain management index (PMI) is a measure of the appropriateness of analgesic therapy [40]. It is a composite measure that reflects the suitability of the strength of the analgesics for a given reported pain severity [41]. Negative PMI values [PMI(−)] indicate undertreatment of pain (inadequate CRP management), while positive PMI values of 0–3 [PMI(+)] are considered to be conservative indicators of acceptable pain treatment [40, 41].

### Ethical considerations

We obtained written and informed consent from all participants. We discussed the aim of the study and its expected outcomes with each patient, and guaranteed the privacy of their data.

### Statistical analysis

We performed revisions and coded the raw data, and utilized Statistical Package for the Social Sciences Version 20 (SPSS, v20) for all our data entry, manipulation, and analysis.

We used descriptive statistics, including means, standard deviations, medians, interquartile ranges (IQRs), and percentages, to summarize patient characteristics. We tested the normality of different variables by applying the Kolmogorov-Smirnov and Shapiro-Wilk tests. For parametric variables, we compared continuous variables of repeated observations using paired t-tests for two groups, and one-way ANOVA for more than two groups. For non-parametric distributions, we used the Wilcoxon signed-rank test to compare two quantitative variables, and the Friedman’s test to compare more than two quantitative variables.

We compared the categorical variables using either Chi-square tests or Fisher’s exact tests, when appropriate. To ease comparisons, we focused on comparing the ESAS-r scores from D6 to the D0 baseline. Supplementary file 1 describes the other time points (D3, D14).

## Results

### Patient demographics and characteristics

**Fig. 1:**
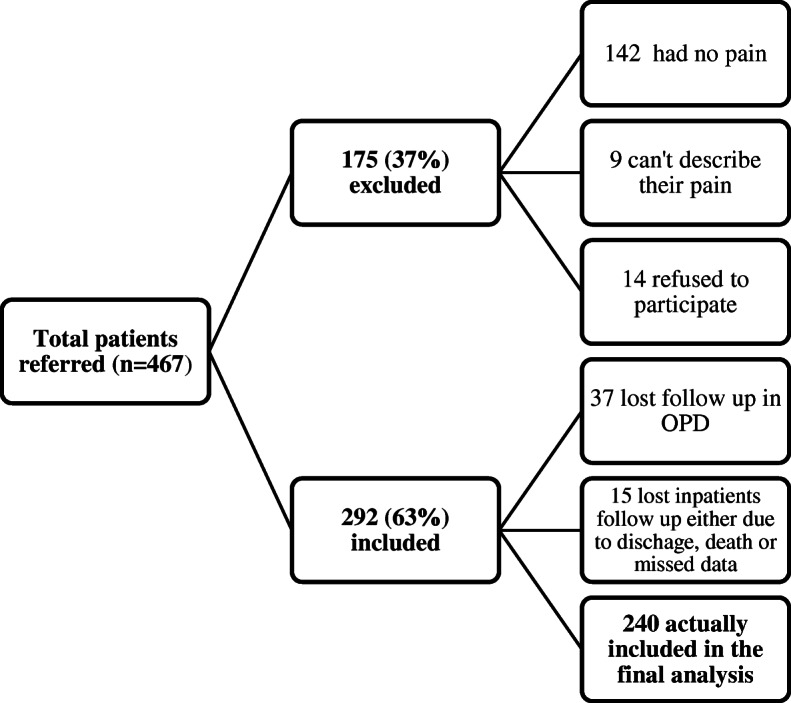
Flow chart of patient eligibility

A total of 467 newly-referred patients arrived at the PCC during the study. We excluded 175 (37%) patients because they did not have pain, could not report their pain, or refused to participate, and we lost 52 (21.67%) patients during follow-ups to the study. Figure 1 provides a flow chart of the patients.

Testing the normality of the ESAS-r scores, opioids and adjuvant medications’ doses and MEDDs with the Kolmogorov-Smirnov and Shapiro-Wilk tests revealed that they had a non-parametric distribution. We used the Wilcoxon signed-rank test to compare two quantitative variables, and Friedman’s test to compare more than two quantitative variables. Other variables, such as age, were normally distributed.

The analysis included two hundred and forty patients. There were 122 (50.8%) men and 118 (49.2%) women. Most of the patients were Kuwaiti (*n* = 114, 47.5%). The most common cancers were gastrointestinal (*n* = 107, 44.58%) followed by thoracic (*n* = 42, 17.5%), genitourinary (*n* = 33, 13.75%), breast (*n* = 26, 10.83%), and head and neck cancer (*n* = 12, 5%). Overall, 85.4% (*n* = 205) of the patients had distant metastasis, especially in their livers (*n* = 119, 49.6%), lungs (*n* = 100, 41.7%), bones (*n* = 81, 33.8%), brains (*n* = 32, 13.3%), and adrenal glands (*n* = 7, 2.9%). A total of 209 (87.1%) patients were referred to us after declarations from their oncologists for best supportive care, and only 31 (12.9%) were still actively on anti-cancer treatment. Only six (2.5%) patients had curative intent (localized disease with no metastasis), while 25 (10.4%) had palliative intent (locally advanced and/or metastatic disease). Inpatients represented 69.6% (*n* = 167) of the total, while outpatients represented 30.4% (*n* = 73). The Kuwait Cancer Control Center was responsible for more than 97% of the referrals to the PCC (*n* = 234), especially in the medical oncology department (*n* = 212, 88.3%) (see Table 1).

**Table 1:** Characteristics of cancer patients referred to Palliative Care Centre with pain in Kuwait

Variables	n (%)
**Age** (mean ± SD)	61.58 ± 13.89
**Sex**	
Males	122(50.8%)
Females	118(49.17%)
**Nationality**	
Kuwaiti	114(47.5%)
Non-Kuwaiti	15(6.2%)
Other Arab^b^	71(29.58%)
Non-Arab^c^	40(16.67%)
**Setting**	
Outpatients	73(30.4%)
Inpatients	167(69.9%)
**Referring hospital**	
KCCC	234(97.5%)
Others	6(2.5%)
**Referring department**	
Medical Oncology	212(88.3%)
Radiotherapy	19(7.9%)
Others	9(3.7%)
**Status**	
Active	31(12.9%)
Best supportive care	209(87.1%)
**Cancer Diagnosis**	
Gastrointestinal	107(44.58%)
Breast	26(10.83%)
Thoracic	42(17.5%)
Genitourinary	33(13.75%)
Head and neck	12(5%)
Others	20(8.33%)
**Metastasis**	
Liver	119(49.6%)
Lung	100(41.7%)
Brain	32(13.3%)
Bone	81(33.8%)
Adrenal	7(2.9%)
**Type of pain**	
Mixed	101(42.1%)
Neuropathic	82(34.2%)
Somatic	17(7.1%)
Visceral	40 (16.7%)
**PMI(−)**^a^	
Before referral	61(25.4%)
Day 6 After Referral	4(1.7%)
**ESAS-r before referral**	
Number of ESAS-r symptoms: median(IQR)	
ESAS-r Pain	8(IQR:7–8)
ESAS-r Tiredness	234(97.5%)
ESAS-r Drowsiness	133(55.4%)
ESAS-r Nausea	170(70.8%)
ESAS-r Lack of appetite	230(95.8%)
ESAS-r Shortness of breath	90(37.5%)
ESAS-r Depression	232(96.7%)
ESAS-r Anxiety	233(97.1%)
ESAS-r Pain	232(96.7%)

n(%): represents patients ‘number and their percent

*KCCC* Kuwait cancer control centre, *ESAS-r* Edmonton symptoms assessment scale-revised, *PMI(−)* Pain Management Index, *IQR* Interquartile range

^a^ *P* value < 0.001 for the difference in PMI before and Day 6 After referral

^b^Such as Egyptians, Syrians, Saudi, Jordanians, Lebanese, Iraqi, Palestinians, others

^c^Such as Indians, Philippines, Pakistanis, Bangladeshis, Iranians, others

### Pain and other symptoms

Most patients described their CRP as mixed pain (*n* = 101, 42.1%), followed by neuropathic pain (*n* = 82, 34.2%), visceral pain (*n* = 40, 16.7%), and somatic pain (*n* = 17, 7.1%) (Table 1).

At D0, the median pain score was 6 (IQR: 4–8), ranging from 3 to 10 (see Table 2). Most of the patients had moderate pain (*n* = 143, 59.6%), nearly one-third had severe pain (*n* = 75, 31.2%), and only 9.2% (*n* = 22) had mild pain (see Table 3). Besides pain, the next most commonly associated symptoms were fatigue (*n* = 234, 97.5%), anxiety (*n* = 233, 97.1%), depression, and worsening of well-being (both *n* = 232, 96.7%). Before referral, most patients had 8 (IQR: 7–8) symptoms by ESAS-r (ESAS-r score > 0 for each symptom, see Table 1).

**Table 2:** Comparison between ESAS-r scores, opioid/non opioid analgesics and adjuvant medications before (D0) and after referral to Palliative Care Centre between D0 and D6

	Before	D6	Z score^**a**^	***P*** value^*^
**ESAS-r pain**	6(4–8)	3(0–4)	− 12.637	< 0.001
**ESAS-r tiredness**	2(2–5)	2(1–4)	−1.936	0.026
**ESAS-r drowsiness**	1(1–6)	1(1–4)	−1.223	0.111
**ESAS-r nausea**	2(1–7)	1(0–2)	−8.105	< 0.001
**ESAS-r lack of appetite**	5(2–8)	3(1–5)	−6.265	< 0.001
**ESAS-r shortness of breath**	2(1–6)	0(0–1)	−2.822	0.005
**ESAS-r depression**	3(2–6)	2(1–4)	−4.842	< 0.001
**ESAS-r anxiety**	6(4–9)	2(1–4)	−5.570	< 0.001
**ESAS-r wellbeing**	6(2–8)	4(3–6)	−8.970	< 0.001
**Opioids/non-opioids analgesics number**				
None	23(9.6%)	0(0%)		
One drug	137(57.1%)	189(78.8%)		
Two drugs	70(29.2%)	46(19.2%)		0.01
Three drugs	8(3.3%)	5(2.1%)		
Four drugs	2(0.8%)	0		
**MEDD in mg/day**				
Regular	60(31–93)	34(22–66)	−3.951	< 0.001
PRN only	8(0–13)	5(2–10)	-2.271	0.027
**Morphine**	49(20.4%)	119(49.6%)		< 0.001
**Oxycodone**	50(20.8%)	32(13.3%)		< 0.001
**Transdermal fentanyl patch**	70(29.2%)	24(10%)		< 0.001
**Tramadol**	69(28.8%)	71(29.6%)		< 0.001
**Paracetamol/NSAIDs Adjuvants**	65(27.3%)	40(16.7%)		0.001
NON	147(61.2%)	38(15.8%)		
One drug	67(27.9%)	76(31.7%)		
Two drugs	15(6.2%)	62(25.8%)	−9.638	< 0.001
Three drugs	9(3.8%)	45(18.8%)		
Four drugs	2(0.8%)	19(7.9%)		
**Gabapentinoids**	57(23.75%)	79(32.9%)		< 0.001
Gabapentin	36 (15%)	57(23.75%)		
	600(300–900)	900(300–1200)	−2.035	0.04
Pregabalin	21(8.75%)	22 (9.15%)		
	300(75–300)	300(150–450)	−0.577	0.564
**Dexamethasone**	13(5.4%)	20(8.3%)		< 0.001
	8(8–16)	8(4–13)	−1.138	0.186
**Antidepressant drugs** n(%) Median (IQR) mg/day				
Amitriptyline	14(5.8%)	44(18.3%)		0.002
	25(10–25)	50(35–100)	−1.931	0.042
Other ADD	15(6.2%)	34(14.1%)		< 0.001
Duloxetine	30(30–60)	60(30–60)	−.587	0.564
Escitalopram	5(5–10)	10(10–10)	−2.333	0.016
Sertraline	0.0	50(50–50)	−15.765	< 0.001
Mirtazapine	15(7.5–15)	15(15–30)	−1.783	0.085
**Benzodiazepines**^b^	10(4.2%)	27(11.2%)		< 0.001
**Antipsychotic drugs**^c^	8(3.3%)	11(4.6%)		< 0.001

*ESAS-r*: Edmonton symptoms assessment scale-revised, ESAS Items represented in median and interquartile range

*MEDD*: morphine equivalent daily dose

n(%):represents patients ‘number and their percent using different opioids and adjuvant medications

Quantitative variables represented as median (IQR)for ESAS-r scores, drug doses

^a^Z score was for Wilcoxon Signed Ranks Test for comparing 2 groups of quantitative data

^b^Benzodiazepines: Midazolam, Lorazepam, Alprazolam, Bromazepam

^c^Antipsychotic drugs: Haloperidol, Quetiapine, Olanzapine, Chlorpromazine

*P value was considered significant if < 0.05

**Table 3:** Detailed prescription of main opioids used in relation to pain severity before and after referral to Palliative Care Centre

		None	Morphine	Oxycodone	TDF	Tramadol
**Before**	Regular	23 (%)	22(9.2%)	24 (10%)	70 (19.2%)	20(8.3%)
	PRN		27(11.2%)	26 (10.8%)	0	49(20.4%)
**Mild**	Regular	5	1(4.5%)	0	7(31.8%)	0
*N* = 22(9.2%)	PRN		0	3(13.6%)	0	2(9.1%)
Moderate	Regular	16(11.19%)	15(10.5%)	12(8.4%)	26(18.2%)	13(9.1%)
*N* = 143(59.6%)	PRN		15(10.5%)	12(8.4%)	0	35(24.5%)
**Severe**	Regular	2(2.67%)	6(8%)	12(16%)	37(49.3%)	7(9.3%)
*N* = 75(31.2%)	PRN		12(16%)	11(14.7%)	0	12(16%)
**Day 3**	Regular	0	53(23.2%)	21(8.8%)	38(15.8%)	27(11.2%)
	PRN	0	61(25.4%)	14(5.8%)	0	59(24.6%)
**Mild**	Regular	0	15(15.2%)	5(5%)	11(11%)	12(12%)
*N* = 100(41.6%)	PRN	0	23(23.2%)	5(5%)		35(35%)
**Moderate**	Regular	0	32(26.7%)	12(10%)	23(19.2%)	12(10%)
*N* = 120(50.0%)	PRN	0	32(26.7%)	8(6.7%)		22(18.3%)
**Severe**	Regular	0	6(30%)	4(20%)	4(20%)	3(15%)
*N* = 20(8.3%)	PRN	0	6(30%)	1(5%)		1(5%)
**Day 6**	Regular	0	58(24.2%)	20(8.3%)	24(10%)	22(9.4%)
	PRN	0	59(25.4%)	12(5%)	0	49(20.4%)
**Mild**	Regular	0	37(39.1%)	8(8.2%)	15(11%)	18(17.6%)
*N* = 168(70%)	PRN	0	43(41.2%)	9(16.2%)	0	39(47.8%)
**Moderate**	Regular	0	18(29%)	10(16.1%)	7(11.3%)	4(6.5%)
*N* = 62(25.8%)	PRN	0	16(25.8%)	1(1.6%)	0	10(16.8)
**Severe**	Regular	0	3(30%)	2(20%)	2(20%)	0
*N* = 10(4.2%)	PRN	0	2(20%)	2(20%)	0	0
**Day 14**	Regular	0	63(25.4%)	19(7.9%)	22(9.2%)	15(6.2%)
	PRN	0	61(26.2%)	8(303%)	0	37(15.4%)
**Mild**	Regular	0	55(51.7%)	11(8.8%)	17(16.2%)	14(12.2%)
*N* = 209(87%)	PRN	0	55(51.7%)	7(7.1%)	0	32(27.4%)
**Moderate**	Regular	0	7(26.9%)	6(23.1%)	4(15.4%)	1(3.8%)
*N* = 26(10.8%)	PRN	0	6(23.1%)	4(3%)	0	5(19.2%)
**Severe**	Regular	0	1(1.6%)	2(40%)	1(20%)	0
*N* = 5(2.1%)	PRN	0	0	0	0	0

**Fig. 2:**
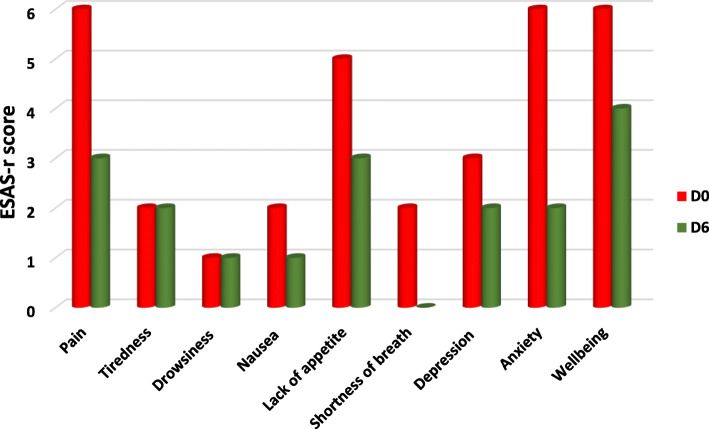
Comparisons of ESAS-r Scores before (Day 0) and After Referral (Day 6) to Palliative Care Centre

After referral to palliative care, patients’ pain and other symptom scores decreased significantly. Pain, anxiety, and depression scores all improved statistically significantly from D0 to D6 [median (IQR): 6 (4–8) to 3 (1–4); 6 (4–9) to 2 (1–4); 3 (2–6) to 2 (1–4), for D0, D3, and D6, respectively, *p* < 0.001 for all] (see Table 2 and Fig. 2). The ESAS-r score for drowsiness did not increase or change from D0 to D6 (*p* < 0.111), with similar scores on D3 and D14 (Supplementary File 1).

**Fig. 3:**
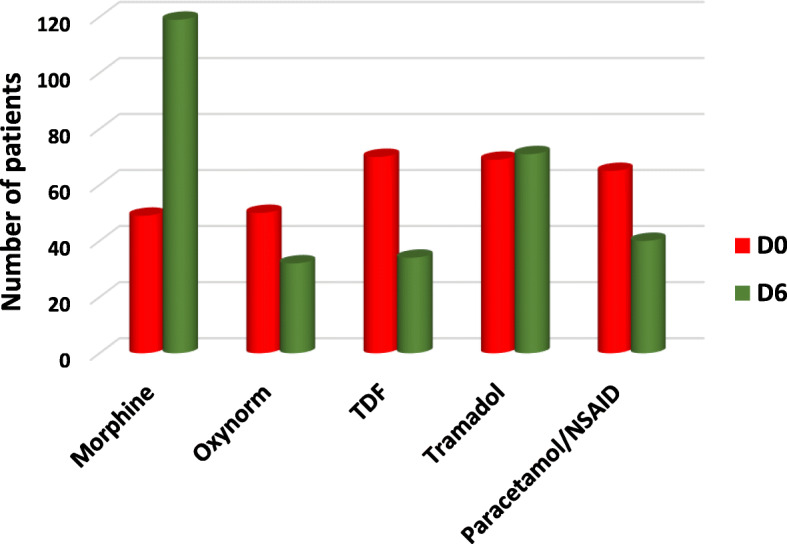
Comparison of different types of opioids used before (Day 0) and after referral (Day 6) to Palliative Care Centre

In comparison to D0, by D6 the number of patients with severe and moderate pain had significantly decreased. There were 75 (31.2%) patients with severe pain on D0 compared to 10 (4.2%) on D6, and 143 (59.6%) patients with moderate pain on D0 compared to 62 (25.3%) on D6, while the number of patients with mild pain increased from 22 (9.2%) to 168 (70%). These results also held for D3 and D14. The improvement in the severity of pain between the days after referral to the PCC was statistically significant from D0 to D3, D6, and D14 (*p* < 0.001 for all; see Tables and Figs. 2 and 3, and Supplementary File 1).

Only five patients reported severe pain by D14, although they had not taken any as-needed doses during the previous 24 h. Interestingly, all five patients had depression and anxiety according to the ESAS-r, and the relationship between severity of pain and the presence of depression and anxiety was statistically significant (*p* < 0.001, data not shown).

### Pain medications

Before referral, 16 (11.19%) patients with moderate pain and 2 (2.67%) with severe pain had had no analgesic medications prescribed. After referral, all patients had at least some as-needed analgesic medications (Table 3).

With regards to opioid prescriptions, morphine was the most common opioid prescribed after referral to the PCC. Its use increased from 20.4% (*n* = 49) before referral to 49.6% (*n* = 119) on D6 (*p* < 0.001), while the use of transdermal fentanyl patches decreased from 29.2% (*n* = 70) before referral to 10% (*n* = 24) on D6 (*p* < 0.001). By D6, the second most commonly used opioid was tramadol (29.6%), followed by oxycodone (13.3%) and transdermal fentanyl patch (10%). Before referral, the transdermal fentanyl patch was the most commonly used opioid (29.2%), followed by tramadol (28.8%), oxycodone (20.8%), and morphine (20.4%). These changes in the prescription patterns were highly statistically significant (p < 0.001 for all). Tables 2 and 3 and Fig. 3 provide details on the opioid prescriptions.

Furthermore, there was a statistically significant increase in the patients’ use of adjuvant medications. Before their referrals, 61.2% of the patients were not taking any adjuvant medications but, by D6, 84.2% were receiving at least one adjuvant medication, and this change was statistically significant (*p* < 0.001). Patients were commonly receiving one or two adjuvant medications [76 (31.7%), 62 (25.8%)] (Table 2). Gabapentinoids were the most commonly prescribed adjuvant medications used either before or after referral although it was significantly higher after referral (D0:*n* = 57, 23.75% vs D6:79(32.9%, *p* value < 0.001).

Comparing D0 to D6, the number of patients using amitriptyline dramatically increased from 14 (5.8%) to 44 (18.3%) (p < 0.001), other antidepressant drugs increased from 15 (6.2%) to 34 (14.1%) (p < 0.001), and anxiolytics (benzodiazepines) increased from 10 (4.2%) to 27 (11.2%) (*p* = 0.002). The data for D3 and D14 was similar (See Supplementary File 1). In addition, the doses and the varieties of antidepressant drugs increased. The median doses of commonly used antidepressant drugs also increased. Amitriptyline doses increased significantly, from 25 mg/day in D0 to 50 mg/day in D6. The same pattern held for duloxetine (from 30 to 60 mg/day) and escitalopram (from 5 to 10 mg/day) (See Table 2).

The PCC used the therapeutic range of anti-depressant doses for amitriptyline, selective serotonin reuptake inhibitors, serotonin and norepinephrine reuptake inhibitors (duloxetine), and Alpha-2 adrenergic antagonists (mirtazapine), following standard recommendations for palliative care [39].

With regards to opioid consumption, the median MEDDs decreased from 60 (IQR:31–93) mg/day before referral to 34 (IQR: 22–66) mg/day on D6 for patients on regular opioids (*p* < 0.001), and from 8 (IQR: 0–13) to 5 (IQR: 2–10) mg/day for patients only on as-needed opioids (*p* = 0.027) (see Table 2). In addition, the PMI(−) decreased significantly, from 25.4% before referral to 1.7% on D6 (p < 0.001, see Table 1).

## Discussion

To the best of our knowledge, this is the first study of its kind, not only in Kuwait, but in the world. Since the PCC is the only available and standalone building offering palliative care service in Kuwait, this study is a benchmark study. Covering the period from January 1, 2016 to December 30, 2017, the study describes cancer patients with CRP’s actual pain control situations, and the pattern of change in their analgesic and adjuvant medication prescriptions, before and after their referral to the PCC, with regular follow-ups on days 3, 6, and 14.

The scores and severities of the patients’ pain and other symptoms significantly improved after referral to the PCC. The presence of heavy symptom burdens and advanced stages of cancer with no active anti-cancer treatments indicated late referrals to PCC. Despite this challenging situation, the PCC significantly improved patients’ pain and other symptoms scores.

In this study, morphine was the least prescribed opioid before referral; however, morphine was the commonly prescribed opioid after referral, which was consistent with World Health Organization guidelines [5, 6], in which morphine is the most effective and recommended opioid for CRP treatment [42, 43].

After their referral to the PCC, patients’ use of adjuvant medications dramatically increased, and over 80% of patients received at least one drug. The most commonly used drugs were gabapentinoids, followed by amitriptyline and other antidepressants. The increased use of sedating adjuvant medications, such as benzodiazepines and amitriptyline, did not change patients’ ESAS-r scores for drowsiness between D0 and D6, which reflects the judicious use of those medications among the PCC’s patients.

In Kuwait, a wide variety of opioids is readily available. Every cancer patient has direct and legal access to opioids from either the Kuwait Cancer Control Center, (a pain clinic and an oncology clinic), or the PCC, based on their primary doctor’s preference. Before referral, pain clinic services, and occasionally oncologists, were in charge of the patients’ pain management. However, an available pain clinic service may be more concerned with physical pain treatment rather than total pain management, which requires a holistic approach to achieve better outcomes. Moreover, one of the most important differences might be that the PCC team spends more time communicating and educating patients and family members about pain and symptom assessment, and their management plan, than other health care providers [20, 21]. This holistic approach is the key to a more person-centered and integrated approach between health and social care services that assesses people’s need for support in order to improve patient outcomes. The aide-memoire we employed is a tool that supports practitioners who are carrying out assessments through a framework that considers patients’ holistic needs [44].

Lower MEDDs were not demonstrating under treatment of pain in our study; arguably, it indicated more appropriately directed opioid use, since number of patients receiving opioids during the follow-up periods was increased. Similarly, a Korean study also reported pain treatment with low MEDDs (median 60 mg/day) [15]. This better pain control, even with lower MEDDs, may be attributed to the holistic approach of CRP management, including assessment and treatment of “psychosocial pain,” and the use of adjuvant medications in conjunction with opioids. Fortunately, the prevalence of alcoholism and illicit drug abuse among the patients in the study was very low, as these factors lead to higher pain expression and, in turn, the need for higher doses of opionds [45, 46]. In many countries, there are new legal regulations that limit high opioid consumption due to increased rates of drug misuse, abuse, addiction, overdose, and death [47–49]. This highlights the need to reconsider opioid prescriptions, and to stop depending on MEDDs as the sole indicator for better CRP treatment.

The relationship between anxiety, depression, and pain intensity is debatable. Some studies have reported no relationship between psychological distress and pain [50], while a recent study reported that patients with higher anxiety and other symptoms scores had poorer responses to breakthrough opioid analgesics [51]. Anxiety and depression are the most common psychological symptoms in patients with cancer pain [51]. This overlap in their clinical presentations, as well as the overlap in the emotional and sensory regions in the brain associated with pain and those affected by depression and/or anxiety, is evidence for the complex association between pain and psychological symptoms [18, 52]. This emphasizes the need for more global symptom assessment, and not just pain management [29, 32, 53].

In addition, conflicting results have been reported regarding the role of adjuvant medications. In two meta-analyses that examined 11 studies in which opioids were used in more than 80% of patients with CRP, neither study was able to demonstrate any benefits in terms of improved pain scores or reduced opioid doses [23, 24]. On the other hand, many studies have reported statistically significantly better pain scores through a combination of opioids and adjuvant medications, with triple combinations (gabapentinoids and/or antidepressants) performing better than dual combinations [19–21, 54].

Recently, there has been a marked increase in the prescription patterns of psychotropic drugs (anxiolytic, antidepressant, and antipsychotic drugs) [55, 56], especially in patients with advanced diseases who are receiving palliative care [56]. This increase reflects the distress and pain experienced by those patients throughout their care trajectories [54].

Although Kuwait has only recently introduced palliative care services, the palliative care team at the PCC was able to achieve good pain and other symptom control in more than 95% of its patients through a holistic approach. This is supported by the Lancet Oncology Commission’s 2018 discussion about the need to complete the integration of palliative care and oncology in order to promote patient-centered care, improve survival, control symptoms, improve family satisfaction, and increase quality of life [57].

### Strength and limitations

Since the PCC is the only available and standalone building offering palliative care services in Kuwait, this study can be considered to be a national survey that describes the actual pain control situation and pattern of change in all analgesic and adjuvant medication prescriptions before and after referring cancer patients with CRP for a two-year prospective audit.

Many adjuvant medications were used for indications other than pain, so it is difficult to say that every medication in this study was used only for pain relief. A longer follow-up period may be more appropriate in assess the long-term control of pain, other symptoms, and the effect of adjuvant medications. We excluded patients without pain from the study, which may not have allowed for a fair comparison of patients’ characteristics, other symptom prevalence, and severity.

### Recommendations

The results of this study will help researchers to design future clinical trials aimed at improving the understanding the role of adjuvant medications, including anxiolytics and antidepressant drugs, in pain control. Early integration of PCC into oncology practice should be implemented to provide better symptom control and quality of life for cancer patients.

## Conclusion

After their referrals to the PCC, patients’ pain and other symptoms scores decreased significantly, even with lower MEDDs, arguably through more appropriately directed opioid use. This is evidence for the effectiveness of the comprehensive program at the Palliative Care Center in Kuwait.

## Supplementary Information

**Additional file 1: Table S1.** Comparison between ESAS-r scores, Opioid/non opioid analgesics and Adjuvant Medications before and after referral to Palliative Care Centre for D0, D13, D14

## Data Availability

The datasets used and/or analysed during the current study are available from the corresponding author on reasonable request.

## Abbreviations

CRP: Cancer-related pain
ESAS-r: Revised Edmonton Symptom Assessment Scale
IQR: Interquartile range
LANSS: Leeds Assessment of Neuropathic Symptoms and Signs
MEDDs: Morphine equivalent daily doses
PCC: Palliative care cancer
PMI: Pain management index
SPSS: Statistical Package for Social Science
