# Supplementary material for: The pattern of change in opioid and adjuvant prescriptions for cancer pain before and after referral to a comprehensive program in the Palliative Care Center in Kuwait
Source: BMC Palliat Care. 2021 Feb 3;20:25. doi: 10.1186/s12904-021-00717-2 (PMC7860188; doi:10.1186/s12904-021-00717-2)
Supplement: Supplementary file 1 — Additional file 1: Table S1. Comparison between ESAS-r scores, Opioid/non opioid analgesics and Adjuvant Medications before and after referral to Palliative Care Centre for D0, D13, D14 [file 12904_2021_717_MOESM1_ESM.docx]

**Table S1: Comparison between ESAS-r scores, Opioid/non opioid analgesics and Adjuvant Medications before and after referral to Palliative Care Centre for D0, D13, D14**

|  |  | **Before** | **D3** | **D14** | **Chi-Square****^$^** | **P value^*^** |
| --- | --- | --- | --- | --- | --- | --- |
| **ESAS-r Pain** |  | 6(4-8) | 4(3-5) | 2(0-3) | 362.55 | <0.001 |
| **ESAS-r Tiredness** |  | 2(2-5) | 2(1-4) | 2(0-3) | 39.62 | <0.001 |
| **ESAS-r Drowsiness** |  | 1(1-6) | 1(1-5) | 1(0-1) | 13.74 | 0.571 |
| **ESAS-r Nausea** |  | 2(1-7) | 2(1-5) | 0(0-2) | 177.19 | <0.001 |
| **ESAS-r Lack of appetite** | | 5(2-8) | 3(2-5) | 2(0-4) | 105.27 | <0.001 |
| **ESAS-r Shortness of breath** | | 2(1-6) | 0(0-1) | 0(0-1) | 41.95 | <0.001 |
| **ESAS-r Depression** |  | 3(2-6) | 3(2-4) | 2(1-3) | 128.04 | <0.001 |
| **ESAS-r Anxiety** |  | 6(4-9) | 3(2-4) | 2(0-3) | 134.30 | <0.001 |
| **ESAS-r wellbeing** |  | 6(2-8) | 5(2-6) | 3(0-5) | 208.15 | <0.001 |
| **Opioids/non-Opioids**  **Analgesics number** | None | 23(9.6%) | 0(0%) | (0%) |  |  |
|  | One drug | 137(57.1%) | 175(72.92%) | 192(80%) |  |  |
|  | Two drugs | 70(29.2%) | 59(24.6%) | 41(17.1%) |  | <0.001 |
|  | Three drugs | 8(3.3%) | 6(2.5%) | 7(2.9%) |  |  |
|  | Four drugs | 2(0.8%) | 0 | 0 |  |  |
| **MEDD in mg/day** | Regular | 60(31-93) | 35(24-68) | 30(17-64) | 153.85 | <0.001 |
|  | PRN only | 8(0-13) | 5(2.5-10) | 4(2-6) | 66.03 | <0.001 |
| **Morphine** |  | 49(20.4%) | 114(47.5%) | 100(51.6%) |  | <0.001 |
| **Oxycodone** |  | 50(20.8%) | 35(14.6%) | 27(11.2%) |  | <0.001 |
| **Transdermal fentanyl patch** | | 70(29.2%) | 38(15.8%) | 22(9.2%) |  | <0.001 |
| **Tramadol** |  | 69(28.8%) | 86(35.8%) | 52(21.6%) |  | <0.001 |
| **Paracetamol/NSAIDs** | | 65(27.3%) | 32(13.3%) | 33(13.7) |  | 0.001 |
| **Adjuvants** | NON | 147(61.2%) | 51(21.2%) | 54(22.5%) |  |  |
|  | One drug | 67(27.9%) | 83(34.8%) | 58(24.2%) |  |  |
|  | Two drugs | 15(6.2%) | 59(24.6%) | 64(26.7%) |  |  |
|  | Three drugs | 9(3.8%) | 31(12.9%) | 48(20%) |  |  |
|  | Four drugs | 2(0.8%) | 16(6.7%) | 16(6.7%) |  |  |
| **Gabapentinoids** |  | 57(23.75%) | 85(35.4%) | 67(27.9%) |  | <0.001 |
| **Dexamethasone** |  | 13(5.4%) | 15(6.2%) | 48(20%) |  | <0.001 |
| **Antidepressant drugs**^**^ | Amitriptyline | 14(5.8%) | 38(15.8%) | 61(25.4%) |  | 0.002 |
|  | Others | 15(6.2%) | 16(6.6%) | 31(12.9%) |  | <0.001 |
| **Benzodiazepines**^***^ |  | 10(4.2%) | 23(9.6%) | 87(32.5%) |  | <0.001 |
| **Antipsychotic drugs**^****^ | | 8(3.3%) | 13(5.4%) | 57(23.8%) |  | <0.001 |

***^$Ch-Square^*** *^of Friedman test for non-parametric data for repeated measurement for more than 2 groups for quantitative data.^*

*^*P value was considered significant if <0.05^*

***^ESAS-r:^*** *^revised Edmonton symptoms assessment scale, ESAS Items represented in median and interquartile range^*

***^MEDD:^*** *^morphine equivalent daily dose.^*

*^**^* ***^Antidepressant drugs:^*** *^Escitalopram, Duloxetine^****^,^*** *^Sertraline, Mirtazapine, Fluoxetine, Paroxetine^*

***^***^* *^Benzodiazepines^*^:^** *^Midazolam, Lorazepam, Alprazolam, Bromazepam^*

*^****^****^Antipsychotic drugs^****^: Haloperidol, Quetiapine, Olanzapine, Chlorpromazine^*
